# Repair potential of self‐assembling peptide hydrogel in a mouse model of anterior cruciate ligament reconstruction

**DOI:** 10.1002/jeo2.12061

**Published:** 2024-06-19

**Authors:** Keitaro Fujino, Natsuki Yamamoto, Yukiko Yoshimura, Atsushi Yokota, Yoshiaki Hirano, Masashi Neo

**Affiliations:** ^1^ Department of Orthopedic Surgery Osaka Medical and Pharmaceutical University Osaka Japan; ^2^ Department of Chemistry and Materials Engineering, Faculty of Chemistry, Materials, and Bioengineering Kansai University Osaka Japan

**Keywords:** anterior cruciate ligament reconstruction, enthesis, RGDS, scaffold, self‐assembling peptide hydrogel

## Abstract

**Purpose:**

Establishing zonal tendon‐to‐bone attachment could accelerate the anterior cruciate ligament reconstruction (ACLR) rehabilitation schedule and facilitate an earlier return to sports. KI24RGDS is a self‐assembling peptide hydrogel scaffold (SAPS) with the RGDS amino acid sequence. This study aimed to elucidate the therapeutic potential of KI24RGDS in facilitating zonal tendon‐to‐bone attachment after ACLR.

**Methods:**

Sixty‐four C57BL/6 mice were divided into the ACLR + SAPS and ACLR groups. ACLR was performed using the tail tendon. To assess the maturation of tendon‐to‐bone attachment, we quantified the area of mineralized fibrocartilage (MFC) in the tendon graft with demeclocycline. Immunofluorescence staining of α‐smooth muscle actin (α‐SMA) was performed to evaluate progenitor cell proliferation. The strength of tendon‐to‐bone attachment was evaluated using a pull‐out test.

**Results:**

The MFC and maximum failure load in the ACLR + SAPS group were remarkably higher than in the ACLR group on Day 14. However, no significant difference was observed between the two groups on Day 28. The number of α‐SMA‐positive cells in the tendon graft was highest on Day 7 after ACLR in both the groups and was significantly higher in the ACLR + SAPS group than in the ACLR group.

**Conclusion:**

This study highlighted the latent healing potential of KI24RGDS in facilitating early‐stage zonal attachment of tendon grafts and bone tunnels post‐ACLR. These findings may expedite rehabilitation protocols and shorten the timeline for returning to sports.

**Level of Evidence:**

Not applicable.

**Abbreviations**
ACL: acute anterior cruciate ligament
ACLR: acute anterior cruciate ligament reconstruction
DMF: *N*, *N*‐dimethylformamide
DMT‐MM: 4‐(4,6‐dimethoxy‐1,3,5‐triazin‐2‐yl)‐4‐methylmorpholinium chloride
FITC: fluorescein isothiocyanate
MFC: mineralized fibrocartilage area
NMM: *N*‐methylmorphiline
PBS: phosphate‐buffered saline
SAPS: self‐assembling peptide hydrogel scaffold
SMA: smooth muscle actin

## INTRODUCTION

Acute anterior cruciate ligament (ACL) injury is one of the most common injuries in sports and exercise. Most patients with ACL injury are athletes and young adults who are likely to undergo ACL reconstruction (ACLR) [2, 29, 31]. ACLR aims to restore knee function to the pre‐injury level and enable a return to sports as soon as possible. However, the time taken to return to sports is generally 6–12 months postoperation, and the secondary ipsilateral ACL injury rate is 7% [9, 14, 42].

Zonal attachment between the tendon graft and bone tunnel is an important factor for successful ACLR [27, 38, 39]. However, the healing process is slow, and the established attachment is disorganized, with poor mechanical properties compared to the native ACL [13, 22]. It takes over 10 months for the attachment to mature after ACLR in humans; in some cases, the tendon grafts fail to incorporate into the bone tunnel [28]. Hence, a rehabilitation protocol is required to allow slow progress and prevent inhibition of the incorporation [39]. Enhancing the establishment of zonal attachment could accelerate the ACLR rehabilitation schedule and facilitate earlier return to sports [3, 16]. Several surgical methods and basic scientific strategies have been investigated in animal and clinical studies on ACLR; however, the most suitable approach remains unclear.

Recently, self‐assembling peptide hydrogel scaffold (SAPS) has been used to improve healing in various fields [1, 12, 21, 30, 35]. SAPS consists of peptide nanofibers and forms a three‐dimensional structure with a pore diameter ranging from 5 to 200 nm. The SAPS structure is similar to that of the natural extracellular matrix component, which promotes tissue regeneration without cytotoxicity or immune response [12, 21, 35]. We developed KI24RGDS (IKIKIKIKIKRGDSKIKIKIKIKI, where K = lysine, I = isoleucine, R = arginine, G = glycine, D = aspartic acid and S = serine), which is a SAPS with the amino acid sequence RGDS that acts as a cell adhesion peptide. The sequence have often been incorporated into biomaterials to promote cell proliferation and interactions [10, 17]. A research study has shown that KI24RGDS remained in the meniscal lesion for 12 weeks without adverse effects in a rabbit model of meniscal defect and successfully enhanced tissue regeneration [33].

In this study, we elucidated the therapeutic potential of KI24RGDS in facilitating zonal tendon‐to‐bone attachment following ACLR in a murine model. We hypothesized that KI24RGDS remains in the bone tunnel for a long time after ACLR and acts as a scaffold between the tendon graft and bone tunnel, facilitating zonal attachment by promoting progenitor cells.

## MATERIALS AND METHODS

### Synthesis of KI24RGDS

The KI24RGDS peptide was synthesized as described previously [33]. Briefly, Fmoc‐Ile‐Alko‐PEG Resin (Watanabe Chemical Industries Ltd.) was placed in a polypropylene column (PD‐10; GE Healthcare Company), washed thrice with methanol and *N*, *N*‐dimethylformamide (DMF) and stirred in 25% dimethyl sulfoxide/DMF for 30 min. After swelling, the resin was washed six times with DMF. Subsequently, 20% piperidine/DMF was added and the mixture was stirred for 30 min to deprotect the Fmoc group. Three equal volumes of amino acids, 90 μL *N*‐methylmorphiline (NMM) and 0.200 g 4‐(4,6‐dimethoxy‐1,3,5‐triazin‐2‐yl)‐4‐methylmorpholinium chloride were added to the resin. After 3 h of condensation, the resin was washed six times with DMF. The Fmoc group deprotection and condensation reactions were repeated to synthesize the peptide (Ile‐Lys (Boc))_5_‐Arg (Pbf)‐Gly‐Asp (OtBu)‐Ser (tBu)‐(Lys (Boc)‐Ile)_5_ with side‐chain protection and resin. Finally, deprotection of the peptide side‐chain protecting group and cleavage of the resin were performed as follows: 8.50 mL of trifluoroacetic acid, 0.50 mL of thioanisole, 0.50 mL of ultrapure water, 0.25 mL of 1,2‐ethanedithiol and 0.75 g of crystal phenol were mixed and stirred for 200 min. After dialysis, using a dialysis membrane (Biotech CE Tubing MWCO: 100–500 D from Spectrum Laboratories, Pisctaway) with a fractional molecular weight cutoff of 100–500 Da, the crude KI24RGDS peptide was obtained as a white powder via lyophilization. It was purified using preparative high‐performance liquid chromatography.

KI24RGDS peptide (18 mg) was dissolved in 300 μL of ultrapure water to obtain a final concentration of 6 wt%. Peptide hydrogels were prepared by mixing the peptide solution with 37.5 μL 10× phosphate‐buffered saline (PBS) in a syringe, allowing it to stand for 1 h in a cool dark place.

### Preparation of KI24RGDS‐fluorescein isothiocyanate (FITC)

The peptide was synthesized as described above. After condensation and deprotection of Fmoc‐Ile‐OH at residue 24, condensation and deprotection of Fmoc‐Gly‐OH were carried out at residue 3 as a spacer. Then 0.234 g (0.6 mmol) FITC, 90 μL NMM and 0.200 g DMT‐MM were added and stirred. After synthesis, the procedure was followed as described above for the final deprotection and preparation of the peptide hydrogel.

### Animals

Sixty‐four male C57BL/6 mice (12–13 weeks old) were used in this study. The mice were divided into the ACLR group (*n* = 24) and the ACLR + SAPS group (*n* = 40), including the mice in which was used KI24RGDS (*n* = 24) and KI24RGDS‐FITC group (*n* = 16) during ACLR. The right knee was subjected to ACLR in all the animals.

### Surgical procedure

**Figure 1:**
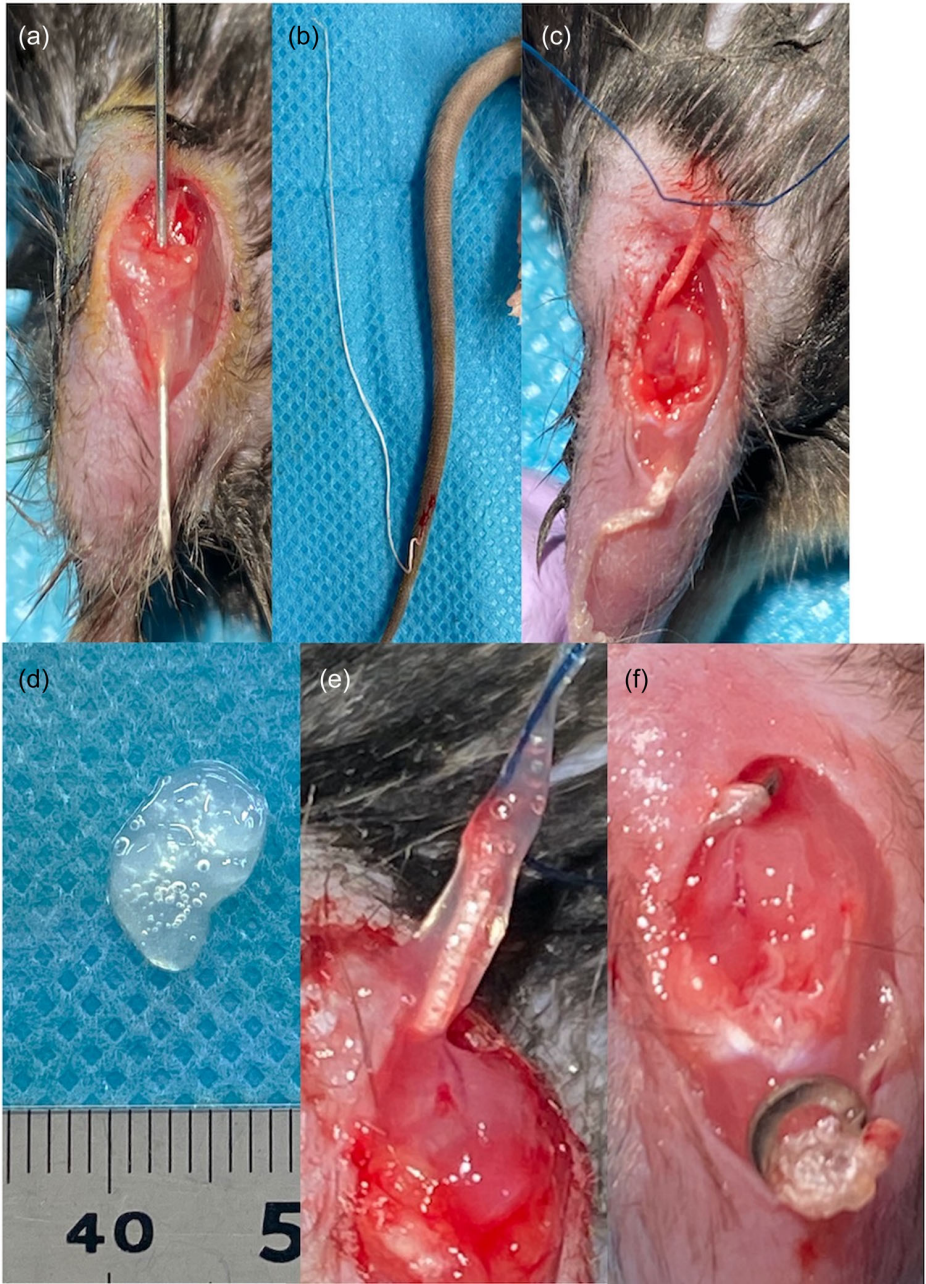
Anterior cruciate ligament reconstruction (ACLR) surgical procedure. (a) The femoral and tibial tunnels were created using a 27G needle after cutting the native ACL. (b) A tendon graft for ACLR was harvested from the tail tendon and it was folded into a double bundle and passed through the tibial and femoral tunnels using a 6‐0 nylon suture (c). (d, e) In the self‐assembling peptide hydrogel scaffold group, 0.5 mL of KI24RGDS was attached to the tendon graft. (f) The tendon graft was fixed using a steel washer at the aperture of the tibial tunnel and exit of the femoral tunnel.

ACLR was performed as described previously [18]. Briefly, anaesthesia was induced with 5% sevoflurane (Maruishi Pharmaceutical) and maintained during surgery with 2.5% sevoflurane. The right knee of each mouse was exposed using an anteromedial incision, and the patella was subluxed. The ACL was transected using a 27G needle at the femur insertion. After confirming anterior instability, tibial and femoral tunnels were made using a 27G needle (Figure 1). The tail tendon was harvested from the same mouse and pulled through the tunnel using a 27G needle and 6‐0 nylon suture. The tail tendon was sutured around a stainless‐steel washer (outer diameter, 2.5 mm; inner diameter, 1.1 mm) at the exit of the femoral tunnel, and then pulled out to the tibial insertion while keeping the knee extended. In the ACLR + SAPS group, SAPS was attached to the tail tendon before getting pulled into the tibial tunnel. Finally, the tail tendon was sutured around the stainless‐steel washer at the tibial tunnel insertion. The patella was then placed back following the medial patellar retinaculum, and the skin was sutured. Animals were allowed unrestricted cage activity after surgery.

### Histological assessment

For temporal evaluation of the right femur, the animals were euthanized on the day after ACLR. The hindlimbs were dissected and fixed in 10% neutral buffered formalin for 2 days, then cryoprotected in 20% sucrose and transferred to 30% sucrose at 4°C overnight. The knees were dissected from the hind limbs and embedded in an embedding medium (SCEM, SECTION‐LAB). Sagittal sections (6 μm thick) were cut parallel to the longitudinal axis of the bone tunnel using a cryostat following Kawamoto's film method [20]. We obtained five sections that showed the longest bone tunnel. The films were attached to microscope slides using a mounting medium (SCMM‐R3, SECTION‐LAB) and then placed under UV light for 5 min. To analyse the histological assessment of tendon‐to‐bone attachment, each film was stained with toluidine blue on postoperative Days 7, 14 and 28. The slides were examined under a light microscope (BZ‐X700; Keyence).

### Labelling and quantifying mineralized fibrocartilage area (MFC)

To assess the maturation of tendon‐to‐bone attachment, we quantified the MFC in the tendon graft 7, 14 and 28 days after ACLR. A day before euthanizing, each mouse was injected intraperitoneally with demeclocycline (30 μg/g; Cayman Chemical) prepared in NaHCO_3_. The sagittal sections parallel to the longitudinal axis of the bone tunnel were examined under a polarizing microscope (Olympus BX‐50; Olympus Corp.). MFC‐labelled demeclocycline in the tendon graft was quantified using ImageJ analysis software (National Institutes of Health). The ratio of MFC to tunnel length was compared between ACLR + SAPS (*n* = 4) and ACLR (*n* = 4) groups on Days 7, 14 and 28 after ACLR.

### KI24RGDS localization and immunohistology

To evaluate the area of KI24RGDS attached to the tendon graft and the change in the area after ACLR on Days 0, 7, 14 and 28, the ACLR + SAPS group (*n* = 4, KI24RGDS‐FITC group) was observed under a fluorescence microscope (BZ‐X700; Keyence). The ratio of the KI24RGDS area to the tunnel area was calculated using ImageJ software.

Next, immunofluorescence staining for α‐smooth muscle actin (α‐SMA) was performed. Transverse sections were washed with PBS for 5 min. Antigen retrieval using 0.1% trypsin (Histofine; Nichirei Corporation) was performed on all sections at 37°C for 20 min and nonspecific binding was blocked with 5% skim milk for 20 min. The sections were incubated with anti‐α‐SMA (1:500, ACTA2; Proteintech) overnight at 4°C to evaluate the proliferation of progenitor cells in the bone marrow [18]. After thorough washing with PBS, the slides were incubated with secondary antibodies conjugated with fluorescence markers (1:100, Alexa Fluor 568; Abcam). Nuclei were stained with DAPI (1:500, DAPI solution; Dojindo). The image was acquired by a fluorescence microscope and co‐stained with DAPI and the α‐SMA cell was examined by ImageJ software. The ratio of co‐stained cells to the total number of cells was calculated and compared between ACLR + SAPS (*n* = 4, KI24RGDS‐FITC group) and ACLR (*n* = 4) groups on Days 7, 14 and 28 after ACLR.

### Biomechanical evaluation

**Figure 2:**
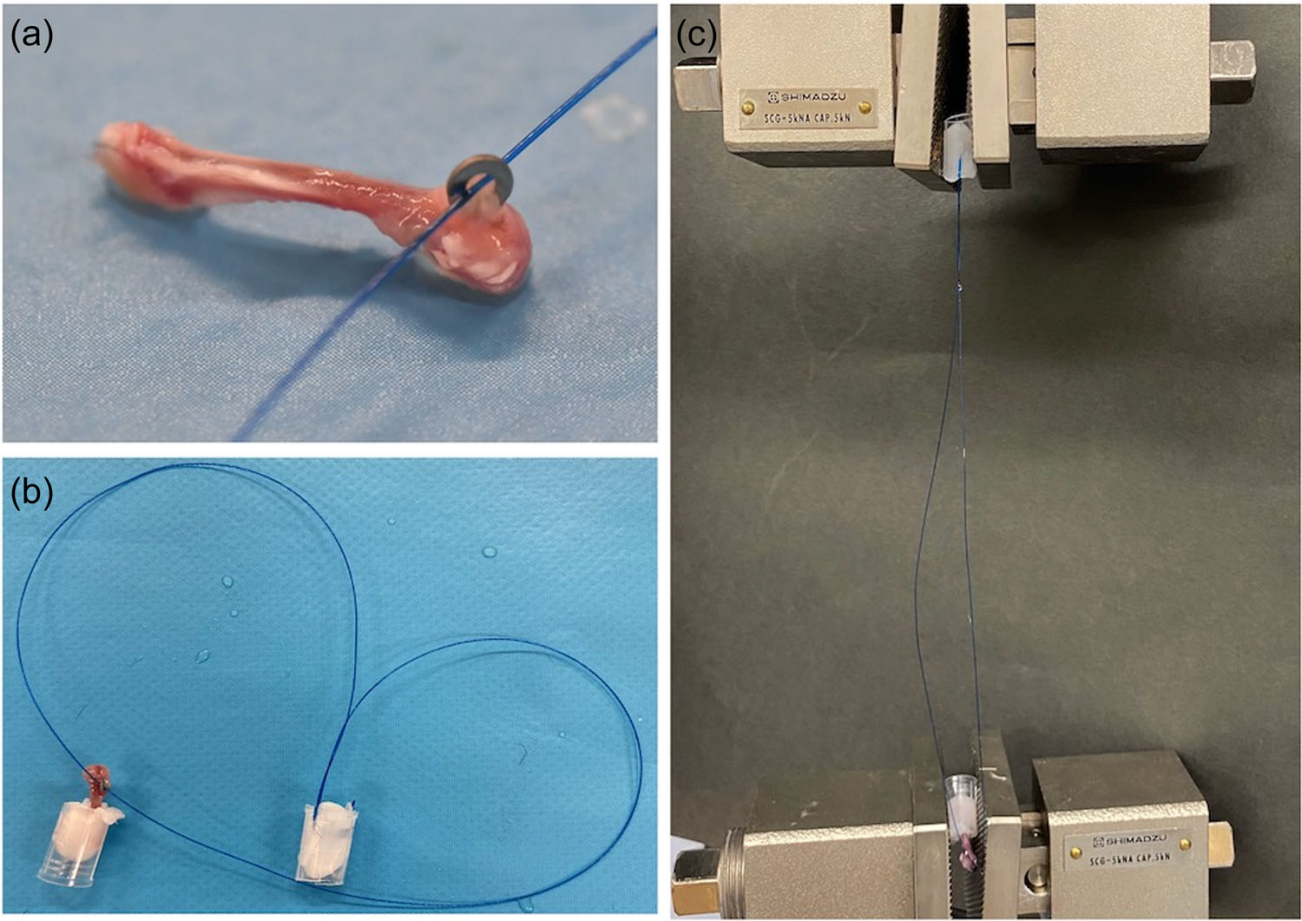
Biomechanical test. (a) Nylon suture passed through the washer of the femur after removal of soft tissue. (b) The proximal femur and tip of the suture were fixed in a plastic case with polycaprolactone. (c) The suture was pulled out towards the tunnel axis until the tendon graft was removed from the bone tunnel.

A pull‐out test was performed to evaluate the failure load of tendon‐to‐bone attachment on Days 14 and 28 after ACLR. Euthanized mice were preserved at −80°C for subsequent biomechanical tests. After thawing, the right femur was dissected and the connective tissue around the femur was removed; however, the washer and tendon graft were retained. A 2‐0 nylon suture was passed through the washer, and the suture end and proximal femur were placed in a plastic pot and fixed with polycaprolactone (Figure 2). The pot was then placed in a universal testing machine (Autograph; Shimadzu Corporation). The suture was pulled parallel to the bone tunnel axis (10 mm/min) until the tendon graft was removed from the bone tunnel. The maximum failure load was calculated and compared between the ACLR + SASP (*n* = 6) and ACLR (*n* = 6) groups.

### Statistical analysis

All results are presented as mean ± standard deviation. The ratio of the KI24RGDS area to the bone tunnel area was analysed using one‐way analysis of variance (ANOVA), followed by post‐hoc analysis using Tukey's test. The ratio of MFC to bone tunnel length, α‐SMA‐positive cells to the total number of cells and maximum failure load were analysed using two‐way ANOVA, followed by post‐hoc analysis with unpaired Student's *t* test or Tukey's test; *p* < 0.05 was considered statistically significant.

## RESULTS

### Histological analysis and MFC quantification

**Figure 3:**
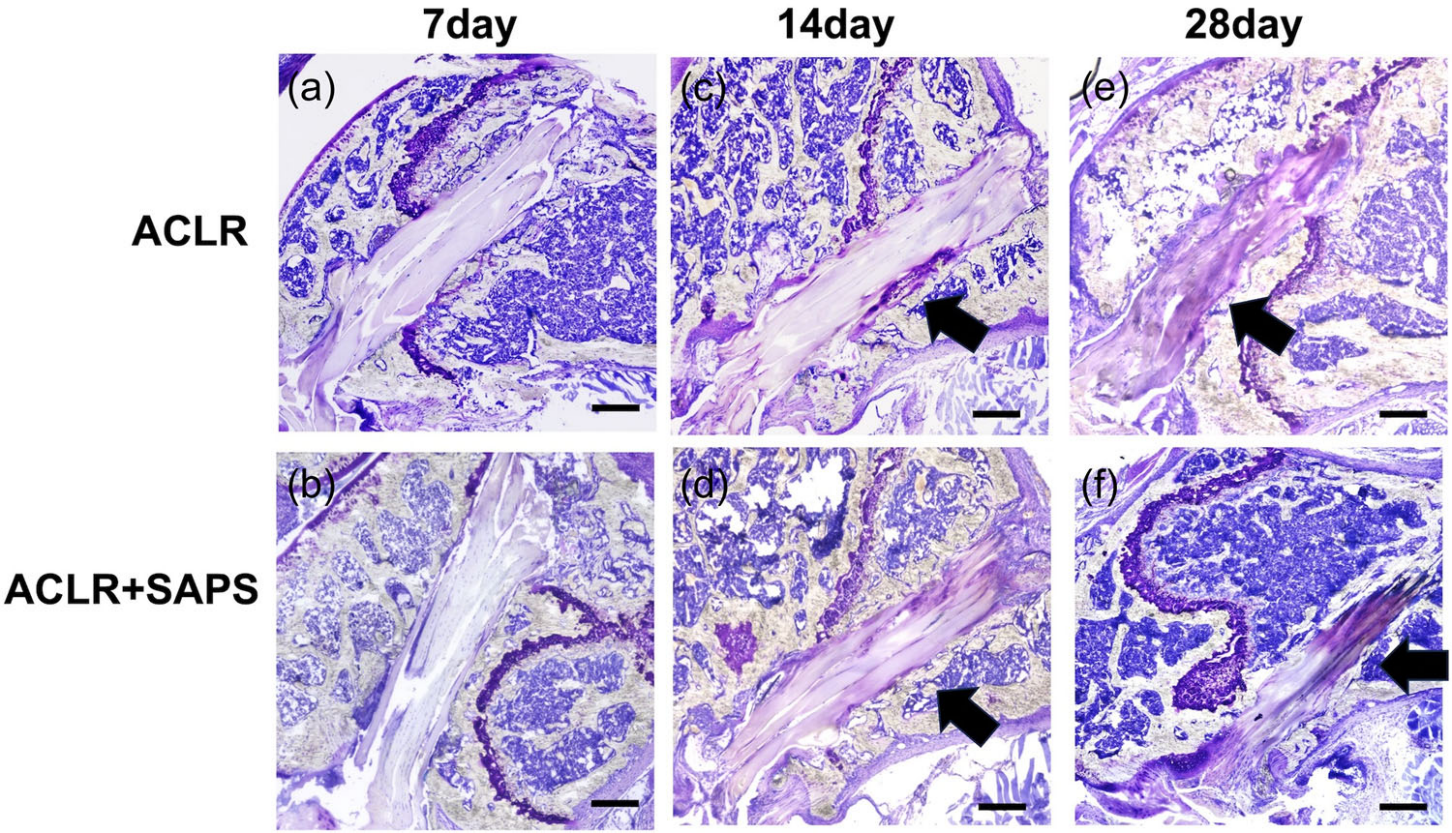
Toluidine blue staining. On Day 7 after surgery, strong proteoglycan staining (indicating fibrocartilage) was not observed in the anterior cruciate ligament reconstruction (ACLR) (a) and ACLR + self‐assembling peptide hydrogel scaffold (SAPS) (b) groups. (c, d) On Day 14, based on the establishment of mineral fibrocartilage, a proteoglycan‐rich area (black arrow) was observed in the tunnel graft in both groups. (e, f) On Day 28, the proteoglycan‐rich area (black arrow) increased more than that on Day 14. Scale bar = 500 μm.

**Figure 4:**
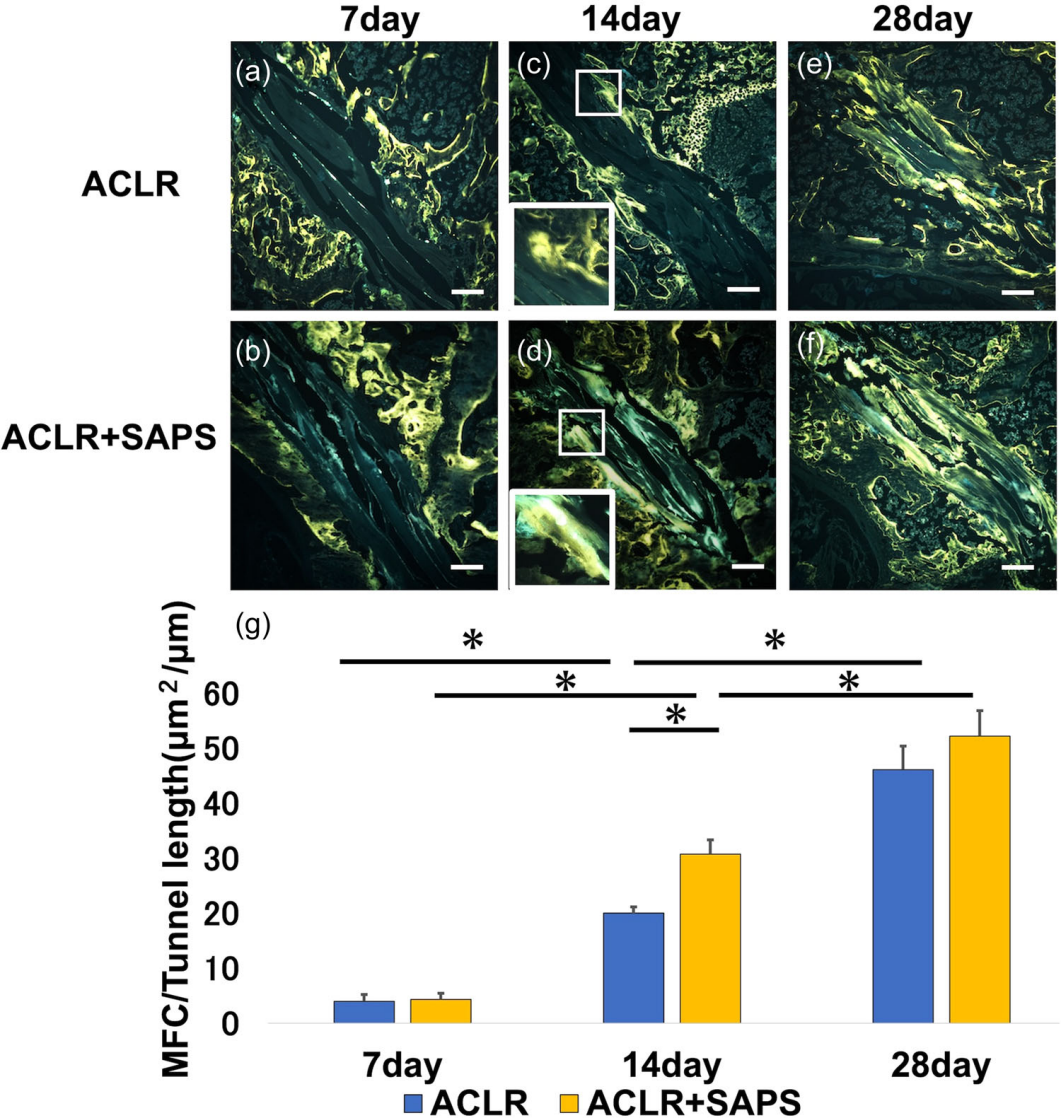
Cryohistology of mineralized tissue. Demeclocycline staining of tendon grafts indicated mineralized fibrocartilage (MFC). On Day 14, the MFC (c, d) between the tendon graft and bone tunnel was significantly increased compared with that on Day 7 (a, b) in both groups. Notably, MFC in the anterior cruciate ligament reconstruction (ACLR) + self‐assembling peptide hydrogel scaffold (SAPS) group was remarkably higher than in the ACLR group on Day 14. (e, f) However, no significant disparity in the ratio was found between the two groups on Day 28 (g). Scale bar = 200 μm. The asterisk indicates a significant difference (*p* < 0.05).

Toluidine blue staining of proteoglycans in the fibrocartilage along with demeclocycline labelling revealed a few MFCs in the tendon graft in both the ACLR and ACLR + SAPS groups on Day 7 post‐ACLR (Figures 3a,b and 4a,b). The ratio of MFC to tunnel length in both groups was 4.0 ± 1.2 and 4.3 ± 1.1 μm^2^/μm, respectively. On Day 14, the partial establishment of MFC was observed in both groups (Figures 3c,d and 4c,d) and the MFC ratio increased compared to that on Day 7 in both the groups (20.0 ± 1.1 and 30.8 ± 2.6 μm^2^/μm, respectively). On Day 28, a large MFC was established between the tendon graft and bone tunnel in both the groups (Figures 3e,f and 4e,f) and the MFC ratio was significantly higher than that on Days 7 and 14 (46.1 ± 4.3 and 52.2 ± 4.6 μm^2^/μm, respectively). On Day 14, the MFC ratio in the ACLR + SASP group was significantly higher than in the ACLR group; however, it was not significantly different on Day 28 (Figure 4g).

### KI24RGDS localization

**Figure 5:**
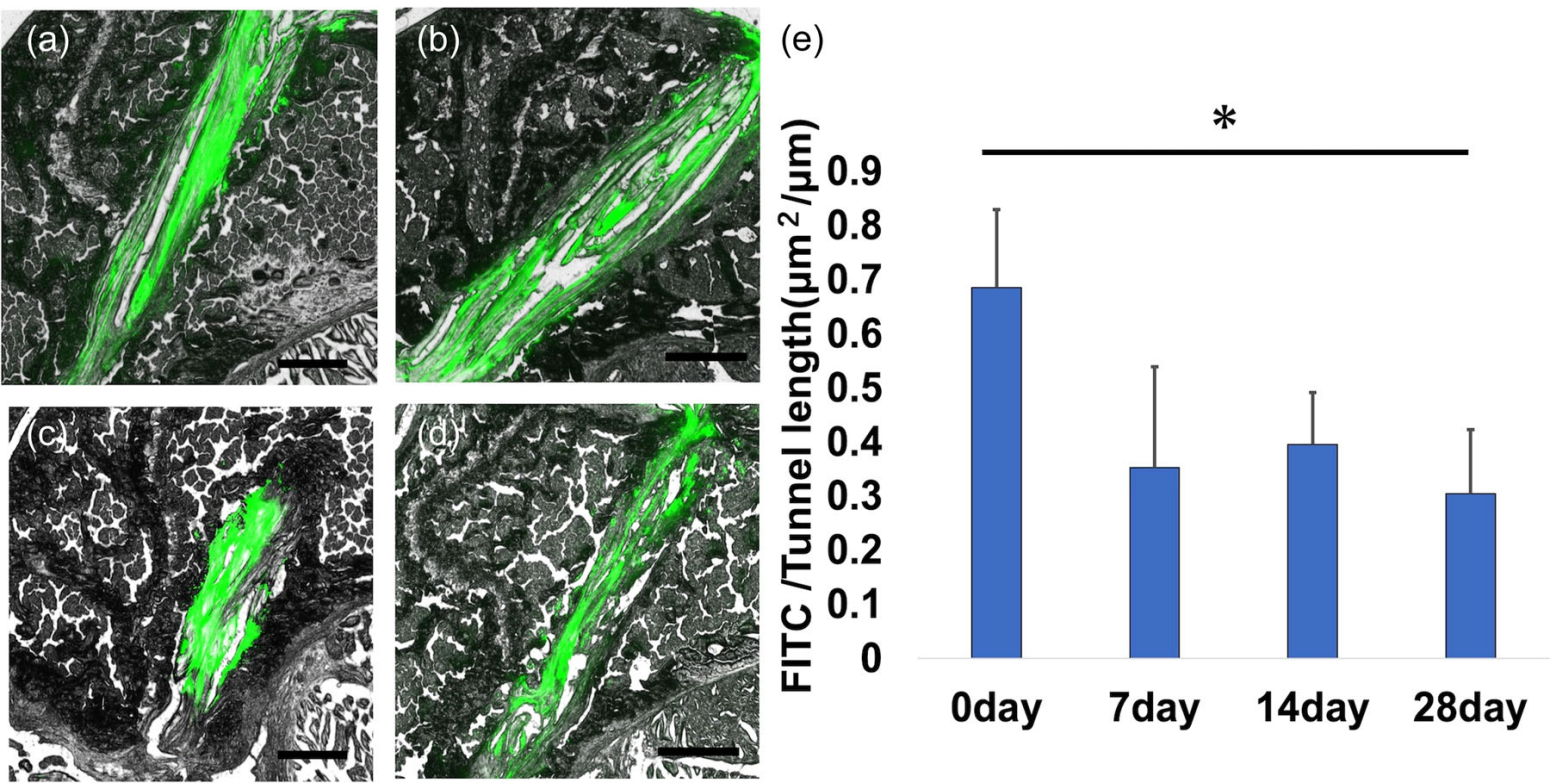
KI24RGDS in the bone tunnel. (a) KI24RGDS attached to the tendon graft filled the gap between the bone tunnel and the graft tendon. (b, c) On Days 7 and 14 after anterior cruciate ligament reconstruction (ACLR), KI24RGDS decreased. On Day 28, the area in the bone tunnel (d) significantly decreased compared to Day 0 (e). However, there was no significant difference between Days 7 and 14. Scale bar = 200 μm. The asterisk indicates a significant difference (*p* < 0.05).

KI24RGDS in the bone tunnel was sufficiently attached to the tendon graft and occupied the space between the tendon graft and bone tunnel on Day 0 after ACLR (Figure 5a); the ratio of FITC area to the bone tunnel area was 68.5 ± 14.4%. On Days 7 and 14, KI24RGDS was clearly decreased in the bone tunnel (Figure 5b,c), although the ratio was not significantly different on Day 0 (35.2 ± 18.7% and 39.6 ± 9.6%, respectively). The KI24RGDS area further decreased on Day 28 (Figure 5d), and the ratio was significantly lower than that on Day 0 (30.4 ± 11.6%), although not significantly different from that on Days 7 and 14 (Figure 5e).

### Immunohistology

**Figure 6:**
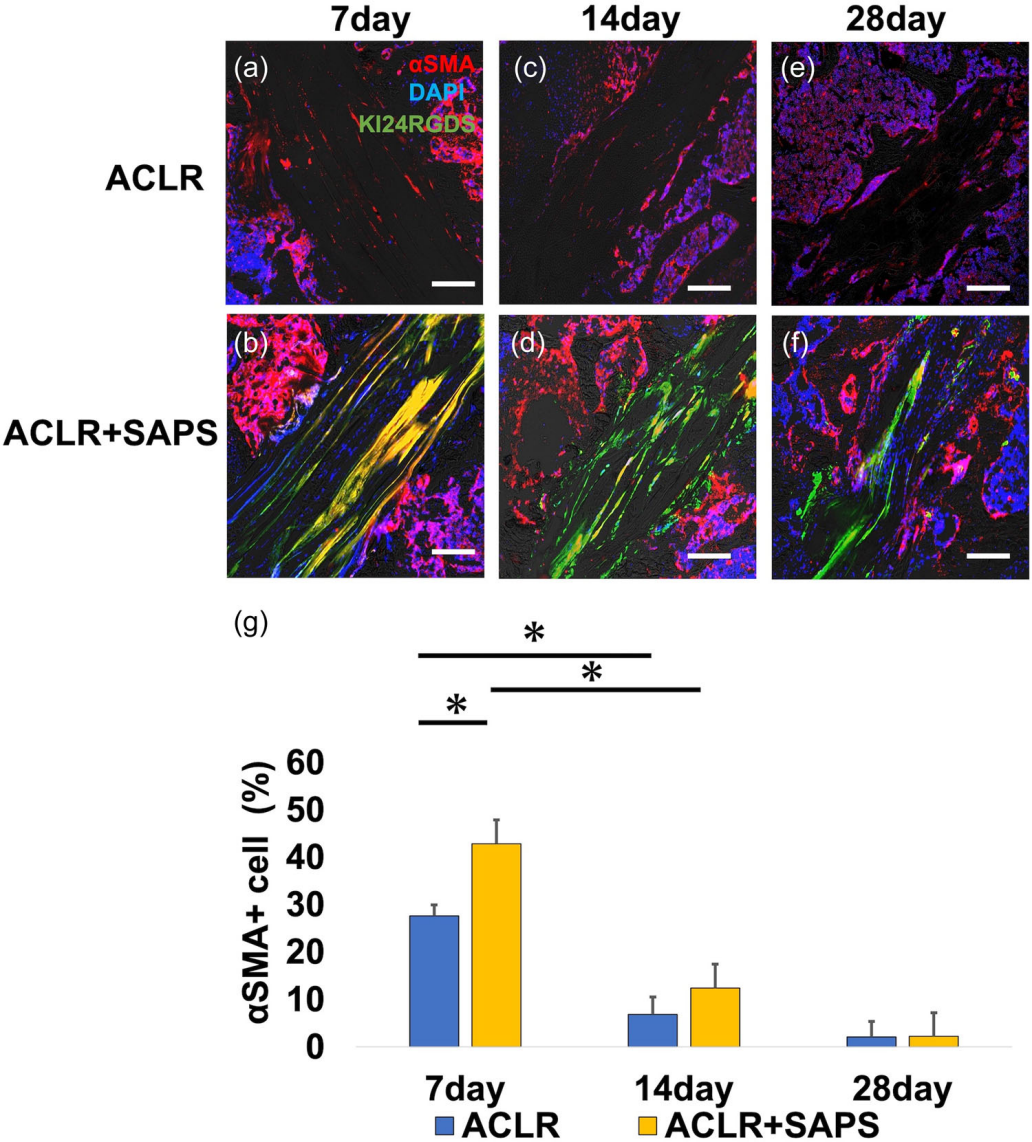
α‐smooth muscle actin (α‐SMA) immunostaining. The number of α‐SMA‐positive cells in the tendon graft was highest on Day 7 after ACLR in the ACLR (a) and ACLR + self‐assembling peptide hydrogel scaffold (SAPS) groups (b). (c, d) On Day 14, the number was remarkably reduced compared to Day 7. (e, f) On Day 30, a few α‐SMA‐positive cells were observed in the bone tunnel. The ratio of α‐SMA‐positive cells to the total number of cells was significantly higher in the ACLR + SAPS group than in the ACLR group on Day 7; (g) however, no significant differences were observed on Days 14 and 28 in both the groups. Scale bar = 200 μm. The asterisk indicates a significant difference (*p* < 0.05).

Numerous α‐SMA‐positive cells were observed in the tendon grafts of both groups on Day 7 after ACLR (Figure 6a,b). In addition, the ratio of α‐SMA‐positive cells to the total number of cells was significantly greater in the ACLR + SAPS group than in the ACLR group (Figures 6g, 42.9 ± 8.4% vs. 27.7 ± 2.3%, respectively). Interestingly, almost all α‐SMA‐positive cells in the ACLR + SAPS group had infiltrated into KI24RGDS. On Day 14, the number of α‐SMA‐positive cells decreased in both the ACLR and ACLR + SAPS groups (Figure 6c,d), and on Day 28, only a few α‐SMA‐positive cells were in the bone tunnel (Figure 6e,f). The ratio of α‐SMA‐positive cells in both the groups on Days 14 and 28 was significantly smaller than that on Day 7 (6.8 ± 3.7% vs. 12.5 ± 4.6%, respectively) and it decreased further on Day 28 (2.1 ± 3.3% vs. 2.2 ± 1.2%, respectively).

### Biomechanical analysis

**Figure 7:**
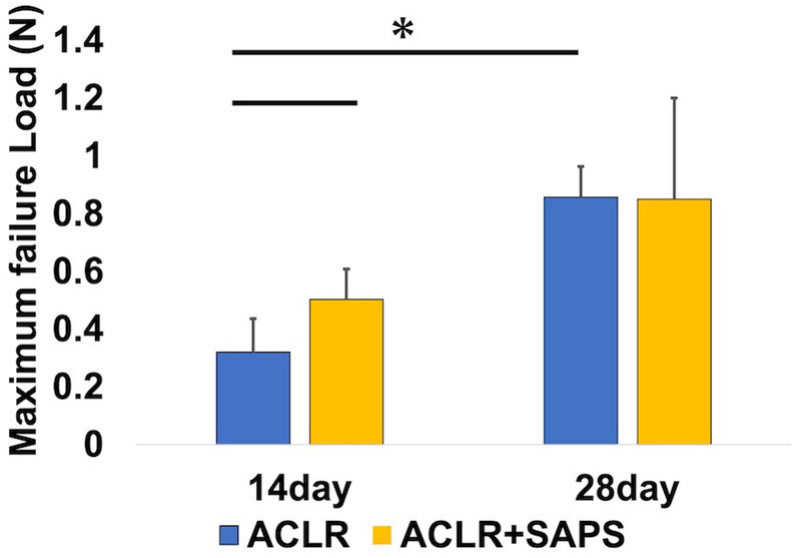
Biomechanical test. Biomechanical analysis indicated that the maximum failure load in the anterior cruciate ligament reconstruction (ACLR) + self‐assembling peptide hydrogel scaffold (SAPS) group was significantly higher than the ACLR group on Day 14 after ACLR. Importantly, in the ACLR group, the maximum failure load was significantly greater on Day 28 than on Day 14 but not in the ACLR + SAPS group. No significant difference was observed between the two groups on Day 28. The asterisk indicates a significant difference (*p* < 0.05).

On Day 14, all tendon grafts were removed from the tunnel in both groups after the pullout force reached the failure load in the mechanical test. Biomechanical analysis showed that the maximum failure load in the ACLR + SAPS group was significantly higher (0.50 ± 0.11 N) than that in the ACLR group (0.32 ± 0.12 N) on Day 14 (Figure 7). On Day 28, most of the tendon grafts were removed from the bone tunnel; however, two tendon grafts in the ACLR + SAPS group were cut at the washer before being removed from the bone tunnel. These samples exhibited a low maximum failure load compared with the other samples. The maximum failure load in both the groups on Day 28 was greater than that on Day 14 (0.86 ± 0.31 N, 0.85 ± 0.35 N, respectively) but was not significant in the ACLR + SAPS group. No significant differences were observed between the two groups on Day 28.

## DISCUSSION

The most important findings in this study were that KI24RGDS promoted the establishment of tendon‐to‐bone attachment at an early stage. In addition, our results suggested that KI24RGDS supports the proliferation of progenitor cells from the bone tunnel to the tendon graft by filling the gap between the two. The rehabilitation protocol after ACLR depends on the establishment of attachment between the tendon graft and bone tunnel. Thus, the use of KI24RGDS in ACLR may accelerate the rehabilitation schedule as well as the return to sports.

However, our results on Day 28 post‐ACLR showed that KI24RGDS did not change the area or mechanical properties of tendon‐to‐bone attachment when compared with ACLR without KI24RGDS, suggesting that KI24RGDS may not lead to a decrease in the reinjury rate, since failure to re‐establish a native insertion and retain biomechanical properties is one of the reasons for the high failure rate of ACLR [28, 34]. Deng et al. demonstrated that the maximum failure load of graft tendon after ACLR in a mouse model was one‐third of native ACL [8]. Several tissue engineering materials have been investigated in clinical and animal studies on ACLR; however, a sufficiently strong tendon‐to‐bone attachment, comparable to that of the native tendon, has not yet been achieved [26, 38]. Although KI24RGDS enhanced the proliferation of progenitor cells, it did not play a role in tendon‐to‐bone establishment. Therefore, the establishment of tendon‐to‐bone attachment depended on a spontaneous healing process. To address this issue, biological enhancement using stem cells or growth factors is likely necessary. Many studies have been conducted on tendon‐to‐bone healing using various types of stem cells, including bone marrow mesenchymal stem cells [24], tendon stem/progenitor cells [25], adipose‐derived stem cells [32] and periosteum‐derived stem cells [5]. These studies have demonstrated that stem cell therapy has enhanced the effectiveness of tendon‐to‐bone healing due to its multipotent and paracrine effects [43]. However, there are challenges such as cell leakage and survival rates when directly transplanting stem cells to a graft tendon or bone tunnel. In a previous study, a combination of tissue engineering and stem cells enhanced the attachment of the mature zonal tendon by removing such disadvantages [11, 43]. As the healing mechanism of the enthesis remains unclear, future studies are warranted to clarify the effect of KI24RGDS with stem cell therapy on ACLR.

The proliferation of progenitor cells and interactions between the tendon graft and bone tunnel are initiated during attachment [18]. However, the width between the tendon graft and bone tunnel is different in each case because the tendon graft is not consistent in size and the drill size for the bone tunnel is limited [29]. This gap slows down the process of establishing zonal attachment. Researchers have demonstrated improvements in the zonal attachment by coating or wrapping tendon grafts with biomaterials, such as chitin [19], hyaluronic acid and gelatin [6], fibronectin [23], collagen matrix [7] and hydroxyapatite‐doped polycaprolactone lactone [15]. However, the optimal material for promoting sufficient regeneration of the zonal attachment remains unclear. This study showed that KI24RGDS has numerous advantages as a scaffold for ACLR. First, its application in clinical settings is easy. KI24RGDS, made from natural amino acids, is a biocompatible and biodegradable biopolymer. SAPS is already in clinical use and its safety is widely recognized. In addition, the synthesis of KI24RGDS can be done at a low cost. Attaching KI24RGDS to the graft tendon during surgery is not difficult. Apart from a refrigerator that can complement the gel, no special equipment or techniques are required. Second, the stiffness and mechanical properties of KI24RGDS make it an appropriate scaffold for ACLR. Okuno et al. demonstrated that KI24RGDS transplanted into rabbit meniscal lesions persisted for 8 weeks after surgery [33]. In this study, we demonstrated that although knee motion was not restricted after ACLR, KI24RGDS filled the gap between the tendon graft and bone tunnel and remained in the bone tunnel for 4 weeks. Third, KI24RGDS is a SAPS with an incorporated amino acid sequence RGDS. The RGDS sequence has been applied to several materials to promote bioactivity and cell adhesion [10, 17]. Some researchers have demonstrated that a scaffold bound to the RGDS sequence enables greater expansion and viability of stem cells than a scaffold without the RGDS sequence [40, 41]. These results suggest that KI24RGDS not only serves as a useful scaffold for promoting cell proliferation but also has the potential to be used as a stem cell‐delivery scaffold.

This study, however, has several limitations. There are some differences between mice and humans. In this study, while 0.4 mm bone tunnels were created in mice, ACLR in humans involves creating bone tunnels of sizes ranging from 7.5–10 mm [36], indicating a significant difference in size. Additionally, it is believed that there are significant differences in the process of tendon remodelling and incorporation between mice and humans [4]. Although in our mouse model we, like many researchers, considered 4 weeks as the endpoint of treatment, in actual clinical practice, a treatment period over 6 months is required [4, 27]. Therefore, there is a possibility that the results of this study may not directly translate to clinical practice. Conducting evaluations of large animals in the future may help address this issue. In clinical practice and studies on large animals, KI24RGDS may not remain in the bone tunnel by the maturation of zonal attachment after ACLR. Therefore, in the future, it may be necessary to adjust the buffer solution concentration and the chain length of the beta‐sheet portion during gel production to allow for the modulation of the denaturation period in vivo. The pull‐out test used in this study evaluated the tendon‐to‐bone attachment site and did not investigate the strength of the graft tendon. Evaluating the strength of the transplanted tendon is also important in clinical practice as the tendon typically fails at the graft midsubstance. However, due to the small size of the mouse knee joint, it was difficult to assess this with our mechanical testing equipment. We believe that conducting experiments on large animals will also help resolve this issue. In addition, we only investigated the femoral tunnel. Further, we compared the histomorphometry parameters with maximum failure load in both the tibial and femoral tunnels. However, we did not investigate the tibial tunnel; we could not perform a reliable mechanical test of the tunnel due to its sharper angle and shorter length as compared with that of the femoral tunnel. The healing process of zonal attachment differs between the tibial and femoral tunnels [37]. However, the healing effects of KI24RGDS on the tibial tunnel remain unclear. Moreover, the failure load in the ACLR + SAPS group on Day 28 may have been lower than the actual value. In a previous study that used a mouse model of ACLR and followed the same surgical procedure, no washer was cut out in the biomechanical test [18]. However, in our study, some KI24RGDS samples were cut at the washer on Day 28. We speculated that KI24RGDS could promote the degeneration of tendon grafts at the washer. Our aims for the future include developing a more reliable and accurate biomechanics test to investigate the biomechanical property of tendon‐to‐bone attachment in the tibia and femur tunnels.

## CONCLUSION

This study highlighted the latent healing potential of KI24RGDS in facilitating early‐stage zonal attachment of tendon grafts with bone tunnels post‐ACLR by promoting progenitor cell proliferation. The findings may expedite rehabilitation protocols and shorten the timeline for return to sports. In addition, KI24RGDS exhibited sufficient biomechanical properties to remain in the bone tunnel for 28 days in a mouse model of ACLR. In the future, taking advantage of the cell proliferative characteristics of KI24RGDS, we plan to investigate the healing potential of KI24RGDS using stem cells to establish a tendon‐to‐bone attachment with even greater similarity to native attachment.

## AUTHOR CONTRIBUTIONS

Keitaro Fujino contributed to the study design, data collection, analysis and writing. Natsuki Yamamoto provided the study material. Yukiko Yoshimura helped with data collection and provided the study material. Atsushi Yokota substantially contributed to the manuscript drafting. Yoshiaki Hirano provided the study material and supervised the project. Masashi Neo supervised the project. All authors critically reviewed and revised the manuscript draft and approved the final version for submission.

## CONFLICT OF INTEREST STATEMENT

The authors declare no conflict of interest.

## ETHICS STATEMENT

All animal procedures were approved by the Institutional Animal Care and Use Committee of Osaka Medical Pharmaceutical University (approval number 22013A).

## Data Availability

The data sets used and/or analysed during the current study are available from the corresponding author upon reasonable request.
